# The copper transport-associated protein Ctr4 can form prion-like epigenetic determinants in *Schizosaccharomyces pombe*

**DOI:** 10.15698/mic2017.01.552

**Published:** 2017-01-02

**Authors:** Theodora Sideri, Yoko Yashiroda, David A. Ellis, María Rodríguez-López, Minoru Yoshida, Mick F. Tuite, Jürg Bähler

**Affiliations:** 1 University College London, Research Department of Genetics, Evolution & Environment and Institute of Healthy Ageing, London, U.K.; 2 Chemical Genetics Laboratory, RIKEN and Chemical Genomics Research Group, RIKEN CSRS, Saitama, Japan.; 3 Kent Fungal Group, University of Kent, School of Biosciences, Canterbury, Kent, U.K.

**Keywords:** yeast, prion, protein aggregation, [PSI+], meiosis, non-Mendelian segregation, oxidative stress

## Abstract

Prions are protein-based infectious entities associated with fatal brain diseases in animals, but also modify a range of host-cell phenotypes in the budding yeast, *Saccharomyces cerevisiae. *Many questions remain about the evolution and biology of prions. Although several functionally distinct prion-forming proteins exist in* S. cerevisiae*, [HET-s] of *Podospora anserina *is the only other known fungal prion. Here we investigated prion-like, protein-based epigenetic transmission in the fission yeast *Schizosaccharomyces pombe*. We show that *S. pombe* cells can support the formation and maintenance of the prion form of the *S. cerevisiae* Sup35 translation factor [*PSI*^+^], and that the formation and propagation of these Sup35 aggregates is inhibited by guanidine hydrochloride, indicating commonalities in prion propagation machineries in these evolutionary diverged yeasts. A proteome-wide screen identified the Ctr4 copper transporter subunit as a putative prion with a predicted prion-like domain. Overexpression of the* ctr4* gene resulted in large Ctr4 protein aggregates that were both detergent and proteinase-K resistant. Cells carrying such [*CTR*^+^] aggregates showed increased sensitivity to oxidative stress, and this phenotype could be transmitted to aggregate-free [*ctr*^-^] cells by transformation with [*CTR*^+^] cell extracts. Moreover, this [*CTR*^+^] phenotype was inherited in a non-Mendelian manner following mating with naïve [*ctr*^-^] cells, but intriguingly the [*CTR*^+^] phenotype was not eliminated by guanidine-hydrochloride treatment. Thus, Ctr4 exhibits multiple features diagnostic of other fungal prions and is the first example of a prion in fission yeast. These findings suggest that transmissible protein-based determinants of traits may be more widespread among fungi.

## INTRODUCTION

Prions were first identified as infectious amyloid forms of the mammalian protein PrP that can be transmitted from organism to organism 1. They were subsequently shown to exist in two fungal species*, Saccharomyces cerevisiae* and *Podospora anserina* 2 and more recently in plants 3. Animal prions are typically associated with catastrophic brain diseases such as Bovine Spongiform Encephalopathy (BSE), human Creutzfeldt-Jakob Disease (CJD) and a variety of other transmissible spongiform encephalopathies (TSEs) 4,5. In each case, these fatal neurodegenerative diseases are associated with refolding of the soluble form of PrP (PrPc) into a distinct conformational state designated PrPSc. The infectious PrPSc conformer can then catalyse the refolding of other PrPc molecules into the PrPSc conformation, which over time leads to amyloid fibrils that form highly ordered aggregates with a characteristic cross β-sheet conformation 6. These amyloid forms are also characteristic of the protein aggregates deposited in the brains of Alzheimer’s, Parkinson’s, and Huntington’s Disease patients. Although the potential for transmission was initially uncovered for prions, recent studies suggest the prion-like spread of a number of other amyloid-based protein aggregates in many neurodegenerative pathologies 7,8,9,10,11,12.

In the budding yeast *S. cerevisiae *there are at least 8 well-established examples of proteins that exhibit prion-like properties 2,13,14,15,16,17, and a systematic survey of the proteome has identified many more potential prion-forming proteins 18. The prion phenomenon is therefore widespread in this yeast species. The most extensively studied *S. cerevisiae* prion is [*PSI*^+^] that is formed by the Sup35 protein, an essential translation termination factor 19,20,21,22. Aside from *S. cerevisiae*, the only other fungal prion so-far established is the [*Het*-s] prion of the filamentous fungus, *Podospora anserina *23. In contrast to their mammalian counterparts, fungal prions do not generally kill their host, although there have been reports of prion-mediated toxicity in *S. cerevisiae* 24,25,26. In most cases, prions in *S. cerevisiae* actually confer a selective growth advantage in a variety of potentially detrimental environments in both laboratory-bred 20,22,27,28 and non-domesticated strains 29.

Budding yeast prions share a number of properties with mammalian prions: they consist of protein aggregates resistant to detergents and proteases, most likely amyloid in nature; they are transmissible without any direct nucleic acid involvement; and overexpression of the soluble protein results in elevated *de novo* formation of ‘infectious’ prion aggregates 30. Besides the fungal and animal prions so far identified and verified, there have also been several recent reports of prion-like mechanisms in mammalian cells 31,32. In fission yeast*,* a ‘prion-like state’ has been reported which allows cells to survive without calnexin and has been linked to an extrachromosomally-inherited determinant designated [*Cin^+^*] 33. It remains to be established whether [*Cin^+^*] is a *bona fide* prion.

The extensive study of *S. cerevisiae* prions has provided crucial information on their mode of propagation, cellular function, and evolution and established prions as a unique class of protein-based epigenetic elements that can have a wide variety of impacts on the host 14,15,16,17,20,22,29,34,35. These studies have also allowed us to define molecular features of prions. All bar two of the verified prions of *S. cerevisiae* contain a discrete prion-forming domain (PrD), a region typically rich in Gln and Asn residues and which is essential for prion formation and continued propagation 2. The exceptions lacking a typical PrD are the Mod5 protein, which confers resistance to antifungal drugs in its [*MOD*^+^] prion state 36 and the Pma1/Std1 proteins that define the [*GAR*^+^] prion 37. Identification of new fungal prion-forming proteins in evolutionarily diverged species can contribute to our understanding of the structure, function and evolution of prions. Notably, while 2.7% of the budding yeast proteins are rich in Gln and Asn residues, only 0.4% and 0.9% of fission yeast and human proteins, respectively, show this characteristic. This bias raises the possibility that fission yeast will offer relevant complementary insight into human prion biology 38.

Fungal prions require specific proteins - molecular chaperones - for their propagation during cell division. In particular, the ATP-driven chaperone Hsp104 is essential for the continued propagation of prions in *S. cerevisiae* 39. Hsp104 breaks aggregates to create additional lower molecular weight seeds (also known as propagons) for prion propagation 37. The chiatropic agent guanidine hydrochloride inhibits the ATPase activity of Hsp104 leading to loss of prions during cell division 40. Although no orthologue of Hsp104 has yet been described in mammals, an orthologue is present in *S. pombe* but was originally reported to be unable to substitute for the *S. cerevisiae* Hsp104 protein in propagation of the [*PSI*^+^] prion in *S. cerevisiae* cells 41. A recent study, however, contradicts this finding by showing that *S. pombe* Hsp104 can indeed substitute for *S. cerevisiae* Hsp104 and propagate *S. cerevisiae* prions 42. This latter study also showed that SpHsp70 (Ssa1 and Ssa2) and the Hsp70 nucleotide exchange factor Fes1 can propagate budding yeast prions, suggesting that *S. pombe* has all of the chaperone machinery used by *S. cerevisiae* to propagate the prion form of several proteins. In neither of these two studies was it established whether this chaperone machinery also plays a role in propagating endogenous prions in *S. pombe*.

In searching for prions in a tractable organism such as *S. pombe*, different criteria can be used to indicate whether or not a specific protein has the ability to form a transmissible prion. These criteria include: (a) overexpression of the soluble protein results in formation of mitotically transmissible aggregates of that protein; (b) the resulting aggregates can be transmitted to cells lacking the aggregates, either naturally by cell fusion (e.g. during sexual reproduction) or experimentally by protein transformation 43; and (c) the phenotype associated with acquisition of the aggregated form of the protein is consistent with a loss of function of the corresponding protein 44.

In evolutionary history, *S. pombe* separated from *S. cerevisiae* over 400 million years ago. Analysing prion behaviour in *S. pombe* could therefore provide a complementary model system to study the establishment and transmission of infectious amyloids and the evolution of prions as epigenetic regulators of host cell phenotypes. Yeast-based models of human amyloidosis have already made important contributions to our understanding of these increasingly prevalent diseases 45,46, but such studies have also revealed differences between the budding and fission yeast models. For example, with respect to α-synuclein amyloids associated with Parkinson’s disease, the E46K α-synuclein mutant is toxic to *S. pombe, *but not to *S. cerevisiae* 43. Yet *S. pombe* has been little exploited in such studies and there is a paucity of tractable model organisms to investigate prion biology. Here, we show that *S. pombe* not only has the cellular machinery to allow a heterologous prion - the [*PSI*^+^] prion from *S. cerevisiae* - to form and propagate, but also has at least one endogenous protein that satisfies the key criteria to define prions with the potential to form a protein-based epigenetic determinant that can impact the phenotype of the host.

## RESULTS

### Fission yeast supports formation of the budding yeast [*PSI*^+^] prion 

**Figure 1:**
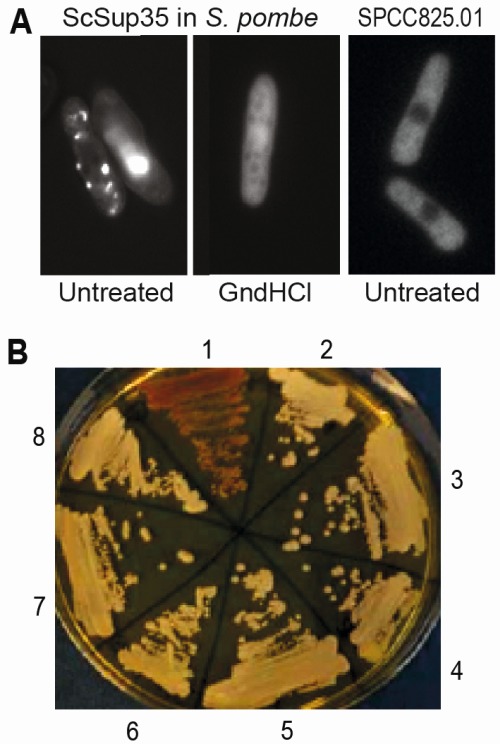
FIGURE 1: Fission yeast can support formation and propagation of the budding yeast [*PSI*^+^] prion. **(A)** Left: Fluorescent foci in *S. pombe *resulting from overexpression of *S. cerevisiae* Sup35-GFP, using the medium-strength, regulatable *nmt41* promoter under activating conditions from a high-copy plasmid. This result resembles the patterns seen when ScSup35-GFP is overexpressed from a high-copy plasmid in *S. cerevisiae *47*.* Middle: The foci are absent from cells grown for 35-40 generations in 3 mM guanidine hydrochloride (GndHCl). Right: Most GFP-tagged *S. pombe *proteins do not show fluorescent foci when overexpressed (see also 54); the uncharacterized protein SPCC825.01(predicted ATPase) serves as an example for such a negative control, showing diffuse cytoplasmic localization. **(B)** Transformation of *S. pombe* cell extract containing ScSup35-GFP aggregates can convert *S. cerevisiae* [*psi*^-^] cells (red, streak 1) to [*PSI*^+^] cells (white, streaks 2-4 and 6-8). Streaks 1 and 5 show control [*psi*^-^] and [*PSI*^+^] strains, respectively.

To test whether *S. pombe* cells can propagate the prion form of a protein, we first tested whether overexpression of the NM region (residues 1 - 254) of the *S. cerevisiae* Sup35 protein (ScSup35) fused to GFP resulted in the generation of heritable protein aggregates. Approximately 20% of cells overexpressing ScSup35 contained either one large or several smaller fluorescent foci consistent with ScSup35-GFP aggregation, with the remaining cells showing diffused cellular fluorescence (**Figure 1A**). This result is similar to the behaviour seen when this construct is overexpressed in *S. cerevisiae* [*PIN*^+^] cells 47. To establish the dependency of the observed ScSup35-GFP aggregation and transmission on Hsp104, cells overexpressing ScSup35-GFP were grown in the presence of 3 mM guanidine hydrochloride (GdnHCl) for 35-40 generations. This treatment completely abolished the formation of fluorescent foci with fluorescence becoming diffused in all cells (**Figure 1A**), indicating that the ScSup35-GFP remained soluble. This ‘curing’ effect of GdnHCl is seen with the majority of prions in *S. cerevisiae* 48, because their propagation is absolutely dependent on Hsp104 activity.

The finding that ScSup35-GFP formed aggregates in *S. pombe* cannot be taken as evidence that these aggregates act as transmissible prions. To test whether the observed aggregates show such prion-like behaviour and can establish a [*PSI*^+^] state, we co-transformed *S. cerevisiae* [*psi*^-^] cells with the pRS416 yeast-centromere plasmid, together with an extract prepared from *S. pombe* cells containing ScSup35-GFP foci. Among 72 *S. cerevisiae *Ura^+^ colonies obtained after transformation, six were confirmed as [*PSI*^+^] colonies by a GdnHCl elimination test (**Figure 1B; Table 1**). This frequency was similar to the one we obtained by co-transformation using a non-sonicated *S. cerevisiae* [*PSI*^+^] cell extract into the same [*psi*^-^] *S. cerevisiae* cells (**Table 1**). Co-transformation with a cell extract prepared from [*psi*^-^] *S. cerevisiae* cells or a *S. pombe* wild-type cell extract gave no [*PSI*^+^] colonies (**Table 1**). Moreover, extracts prepared from *S. pombe *cells containing ScSup35-GFP aggregates, grown in the presence of 3 mM GdnHCl for at least 30 generations, gave no [*PSI*^+^] transformants (**Table 1**), suggesting that inhibition of *S. pombe* Hsp104 prevents the establishment of the transmissible ScSup35-GFP aggregates in *S. pombe*. These results lead us to conclude that fission yeast contains the molecular machinery required for the formation of the [*PSI*^+^] prion with Hsp104 playing an essential role.

**Table 1:** Number of [*PSI*^+^] Ura^+^ *S. cerevisiae* colonies after transformation with cell-free extracts prepared from different species and strains as indicated. *Sc: S. cerevisiae*; *Sp: S. pombe*

**Cell-free extract source**	**Total Ura^+^ colonies**	**[*PSI*^+^] colonies**
*Sc *[*psi*^-^]	75	0
*Sc *[*PSI*^+^]	84	7
*Sp *wild-type	88	0
*Sp *pREP41-ScSup35-GFP	72	6
*Sp *pREP41-ScSup35-GFP, GdnHCl treated	98	0

### Search for prion candidates in fission yeast 

To search for endogenous *S. pombe* proteins that might show prion-like features, we compiled a list of 80 candidate prions based on two sources: proteins identified by mass spectrometry to be insoluble and detergent resistant in [*Cin^+^*] cells, and Q/N-rich proteins identified bioinformatically 38. These candidate proteins were then experimentally tested for prion-like features using a range of assays: 1) overexpression of fluorescently tagged proteins to test for distinct cellular foci; 2) induction of lasting phenotypes upon transient protein overexpression with 40 different environmental and stress conditions being tested; and 3) inheritance of induced phenotypes in a non-Mendelian manner. Unfortunately, none of the 80 candidate proteins showed positive results in all three of these assays and no protein seemed therefore sufficiently promising to further pursue.

After these initial attempts leading to negative results, we applied the PLAAC algorithm that accurately predicts PrDs based on the extensive sequence and functional data from *S. cerevisiae* prion-forming proteins 49. A PLAAC screen of the entire fission yeast proteome identified 295 proteins that contained putative PrDs (**Supplemental Table 1**). Two of these proteins, Fib1 and Myo1, were included among the 80 candidate proteins used in the initial screen. We looked for enriched features among these proteins using the AnGeLi tool 50. The 295 proteins were strongly enriched for Ser, Pro, Asp and Thr residues (p ~9.9 x 10^-12 ^to 0.002) and under-enriched for Lys, Leu, Ile and Glu residues (p ~7.5 x 10^-10 ^to 0.001). Moreover, these proteins were enriched for features diagnostic of plasma membrane and cell surface proteins, including the Pfam domain ‘Ser-Thr-rich glycosyl-phosphatidyl-inositol-anchored membrane family’ (p ~0.0009), GPI anchor surface proteins (p ~0.0007), and the GO cellular component ‘anchored component of external side of plasma membrane’ and related categories (p <0.004).

We performed some initial *in vivo* tests on 30 proteins with high PLAAC scores to identify the most promising prion candidates. Following overexpression of the respective proteins, the cells were subject to a variety of analyses, including assaying an array of growth phenotypes and were also screened for the presence of detergent-resistant forms of the protein using semi-denaturing detergent agarose gel electrophoresis (SDD-AGE).

### Ctr4 contains predicted prion-forming domain in disordered region 

**Figure 2:**
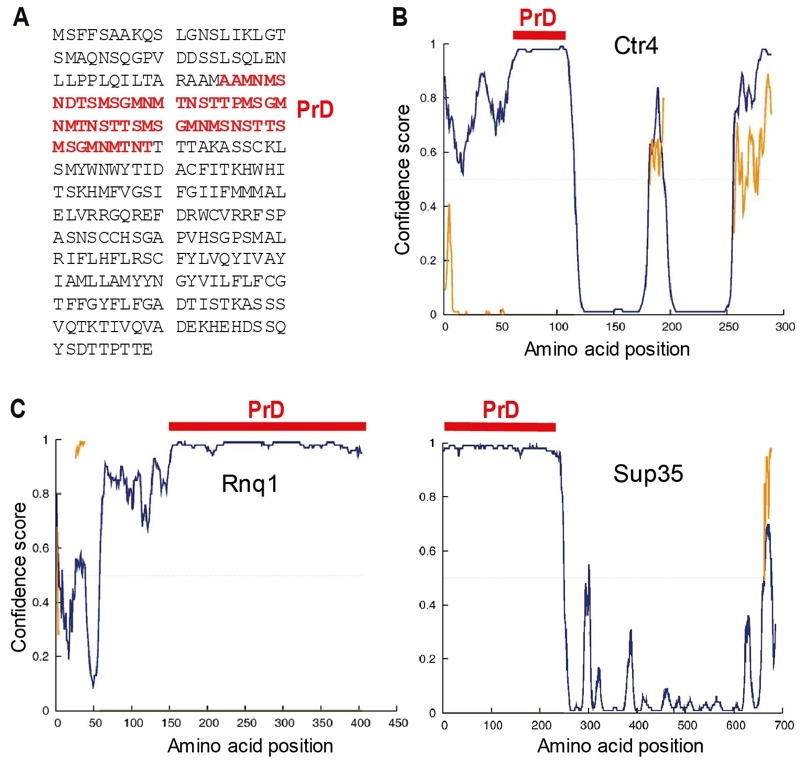
FIGURE 2: Sequence features of Ctr4. **(A)** The 289 amino acid Ctr4 protein contains a 55 amino acid prion-forming domain (PrD, red) as predicted by the PLAAC algorithm 49. **(B) **The predicted PrD of Ctr4 (red bar) coincides with the highest predicted unfolded region (disordered, blue curve) according to the DISOPRED3 algorithm 51. The yellow trace is the location of predicted protein binding sites within disordered regions. **(C)** DISOPRED3 predictions of intrinsically disordered regions in two prion-forming proteins of *S. cerevisiae*, Rnq1 (left) and Sup35 (right), together with the locations of the experimentally defined PrDs, as in (B).

Based on these preliminary analyses, we focused on the Ctr4 copper transporter protein which contains one strongly predicted 55 amino-acid PrD (residues 55-109), consisting of 10 Asn but no Gln residues (**Figure 2A**). Notably, this PrD exactly maps onto an intrinsically disordered region of the protein as predicted by the DISOPRED3 program 51 which identifies residues likely to be natively unfolded (**Figure 2B**). A similar analysis with two established prion-forming proteins of *S. cerevisiae, *namely Sup35 and Rnq1, shows that in both cases a predicted highly disordered region maps to the predicted and functionally defined PrDs of these proteins (**Figure 2C**).

### Ctr4 forms proteinase-resistant polymers

**Figure 3:**
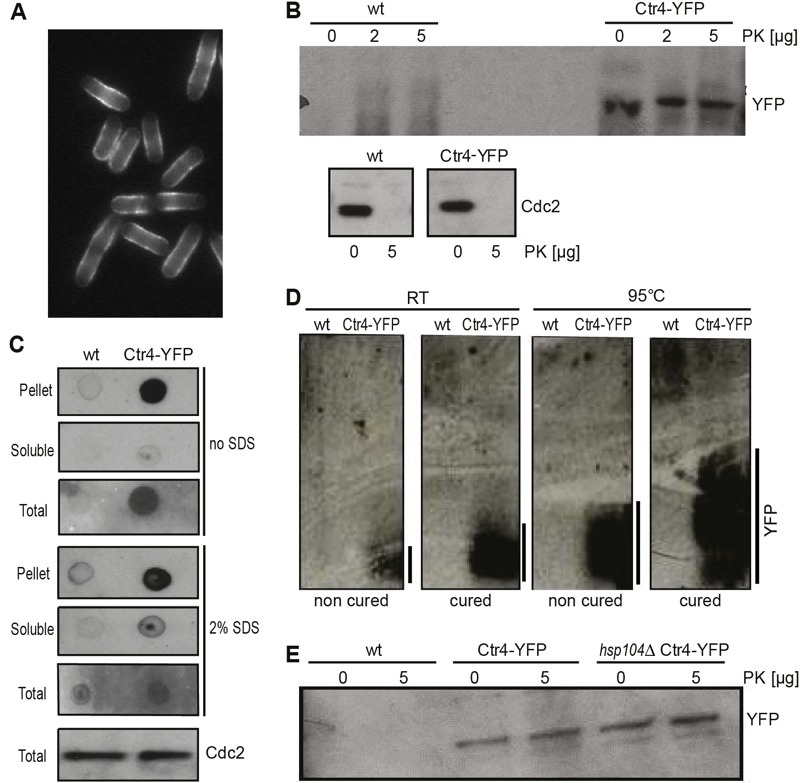
FIGURE 3: Ctr4 exhibits properties consistent with prion formation. **(A)** Fluorescence micrograph of cells overexpressing Ctr4-YFP, showing localization and aggregation of Ctr4 at cell periphery. **(B)** Extracts from wild-type (wt) cells, expressing native Ctr4, and cells overexpressing Ctr4-YFP, treated without (0) or with 2 or 5 µg proteinase K (PK) were run by SDS-PAGE followed by western blotting using anti-GFP and anti-Cdc2 antibodies to detect Ctr4 and Cdc2 (loading control), respectively. **(C)** Dot plots with extracts from wild-type (wt) cells and cells overexpressing Ctr4-YFP using anti-GFP antibodies to detect YFP. Results for pellet, soluble and total cell fractions are shown as indicated, both with and without pre-treatment of the nitrocellulose membranes with 2% SDS. Bottom: SDS-PAGE of the same extracts to detect Cdc2 as a loading control. **(D)** SDD-AGE gels of samples treated at room temperature (RT) and at 95°C, both with and without curing with GdnHCl as indicated. Overexpressed Ctr4-YFP forms high-molecular weight protein aggregates (right lanes); heat treatment and curing did not abolish the high-molecular weight species of Ctr4, but led to a larger range in aggregate sizes. **(E)** Extracts from wild-type (wt) cells, cells overexpressing Ctr4-YFP and *hsp104 *deletion cells overexpressing Ctr4-YFP, treated without or with 5 µg PK were run by SDS-PAGE followed by western blotting using anti-GFP antibody to detect YFP.

To establish whether Ctr4 could switch to a transmissible aggregated state expected for a prion, we first asked whether overexpression of Ctr4 generated aggregates that were resistant to proteinase K (PK) degradation and to detergents such as sodium dodecyl sulphate (SDS) 52,53. Ctr4 was overexpressed using the *nmt1 *promoter driving a full-length genomic copy of *ctr4* fused to YFP, using the corresponding strain from the *S. pombe* ORFeome collection 54. Unlike for ScSup35-GFP (**Figure 1A**), overexpressed Ctr4-YFP did not form distinct cytoplasmic foci, but rather localised to the cell periphery, although Ctr4 clusters and ribbon-like patterns were also evident (**Figure 3A**; 54). This result is in contrast to Ctr4-GFP expressed at lower levels which localizes more evenly to the cell periphery 55.

We explored whether Ctr4-YFP in these cells existed in a different conformational state. Hence extracts from exponentially growing cells overexpressing Ctr4-YFP were treated with PK as described in Materials and Methods, followed by western analysis using an anti-GFP antibody. No or little degradation of Ctr4-YFP by PK was observed in these cell extracts, while Cdc2, a protein not predicted by PLAAC to show prion-like features, was fully degraded by PK (**Figure 3B**). These findings suggest that Ctr4-YFP was indeed in an altered conformational state.

The resistance of the overexpressed Ctr4-YFP to SDS was further assessed using a dot blot assay that has been widely used to analyse disease-associated amyloids 56. First, differential centrifugation was used to fractionate cell-free extracts prepared from cells overexpressing Ctr4-YFP. The resulting insoluble extract (pellet fraction), together with the soluble and unfractionated extracts, were spotted onto nitrocellulose membranes, with or without treatment with 2% SDS for 10 min at room temperature. The bulk of the Ctr4-YFP in these strains was detected in the pellet fraction, and this signal remained strong even following treatment with 2% SDS (**Figure 3C**). These data are consistent with the Ctr4-YFP forming detergent resistant aggregates when overexpressed.

To further explore the nature of the altered conformational form of Ctr4, we exploited semi-SDD-AGE, a method commonly used to detect high-molecular weight, detergent resistant prion aggregates in *S. cerevisiae* 57. We analyzed cell extracts from control and Ctr4 overexpressing cells by SDD-AGE. High-molecular weight protein polymers were only evident in cells overexpressing Ctr4-YFP, and these aggregates were resistant to heat treatment at 95°C (**Figure 3D**). Taken together, these results show that overexpression of Ctr4 results in proteinase- and detergent-resistant protein polymers, consistent with Ctr4 being able to form prion-like structures *in vivo.*

### Hsp104 is not required for the formation or propagation of Ctr4 aggregates 

We next investigated whether the *S. pombe* orthologue of Hsp104 (SpHsp104) is necessary for formation and/or propagation of the Ctr4-YFP aggregates in *S. pombe*. In contrast to most budding yeast prions 58, growth of Ctr4-overexpressing cells in the presence of 3 mM GdnHCl, an inhibitor of Hsp104 ATPase activity, did not abolish Ctr4 polymer formation as judged by SDD-AGE (**Figure 3D**). Furthermore, both heat and GdnHCl treatment increased the size ranges of the Ctr4-YFP aggregates formed (**Figure 3D**).

These results might reflect that SpHsp104 is resistant to GdnHCl as has been reported for the *C. albicans* Hsp104 59, although the finding that the formation of a heterologous prion was blocked by GdnHCl treatment suggests otherwise (**Figure 1A**). To unequivocally define the role of SpHsp104, we deleted the* hsp104* gene from the Ctr4-YFP overexpression strain and repeated the test for PK-resistance of the protein after growing cells for at least 80 generations. Ctr4-YFP remained resistant to PK even in the absence of Hsp104 (**Figure 3E**). This finding indicates that the acquisition of PK-resistant forms of Ctr4-YFP does not require Hsp104, consistent with the result that GdnHCl did not eliminate Ctr4-YFP aggregation (**Figure 3D**). These results therefore suggest that SpHsp104 is not required for Ctr4-YFP aggregation, and raise the possibility that another cellular chaperone may be required for the propagation of the aggregated form of Ctr4-YFP.

### Ctr4 overexpression results in heritable sensitivity to oxidative stress 

Prion-forming proteins in *S. cerevisiae* impact on a wide range of phenotypes when they take up their prion form, often reflecting a loss of function 60. To investigate whether the conformational change seen in cells overexpressing Ctr4 resulted in an altered phenotype, we assayed the growth of the overexpression strain under heavy metal, heat and oxidative stress. While no changes were observed in response to stresses induced by cobalt and heat shock for example (data not shown), the strain did show an increased sensitivity to 2 mM hydrogen peroxide, an inducer of oxidative stress 61.

**Figure 4:**
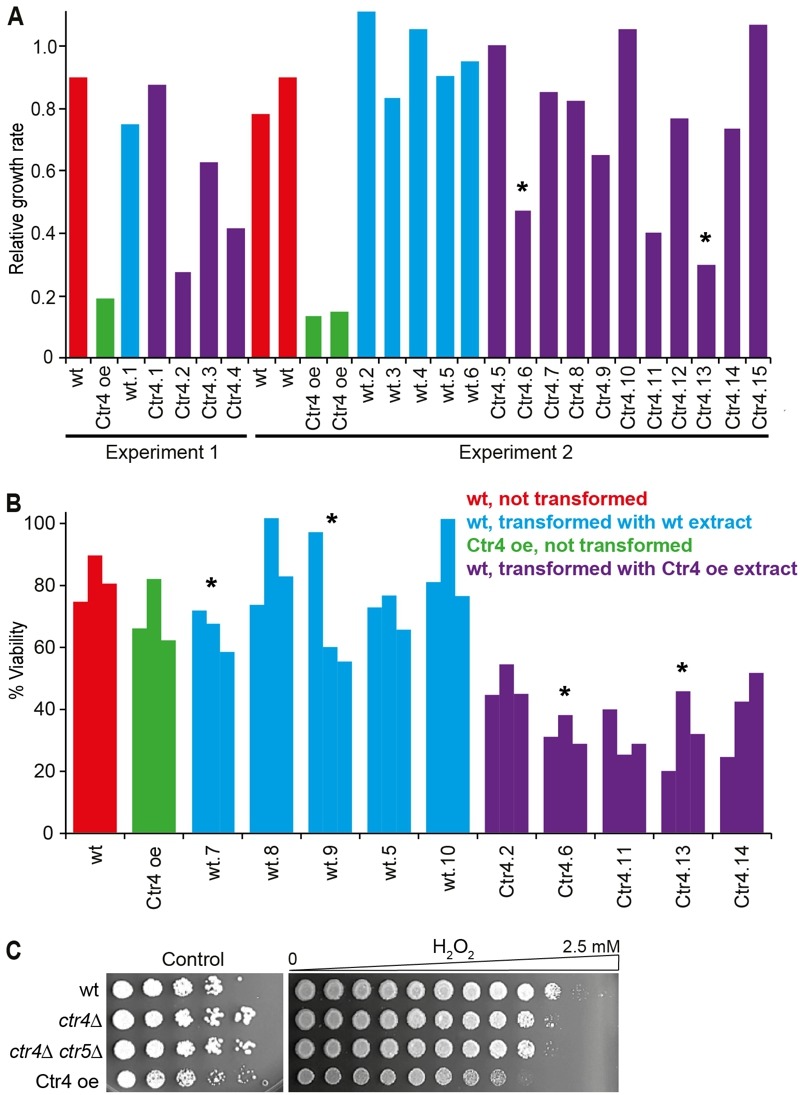
FIGURE 4: Ctr4 overexpression leads to H_2_O_2 _sensitivity which is transmissible by protein transformation. **(A)** Left, Experiment 1: wild-type cells were transformed with a cell-free extract from wild-type (wt.1) and Ctr4 overexpressing cells (Ctr4.1-Ctr4.4). For all strains, the ratios of maximum growth rate in liquid medium with 1 mM H_2_O_2,_ relative to maximum growth rate in untreated medium, were determined in a Biolector microfermentor. Data for control wt and Ctr4 overexpression (Ctr4 oe) cells are also shown. Right, Experiment 2: as Experiment 1, but showing additional, independent transformants with extracts from wild-type (wt.2-wt.6) and Ctr4 overexpressing cells (Ctr4.5-Ctr4.15). Data for two independent wild-type control (wt) and two independent Ctr4 overexpression (Ctr4 oe) cells are also shown. Strains whose extracts were used for the protein transformations in the meiosis experiments (Figure 5) are indicated with asterisks. **(B)** Wild-type cells were transformed with extracts from wild-type (wt.5, wt.7-wt.10) or Ctr4 overexpressing cells (Ctr4.2. Ctr4.6, Ctr4.11, Ctr4.13, Ctr4.14). Cell viability after treatment with 0.5 mM H_2_O_2_ relative to untreated cells was determined for the transformed strains and for control wt and Ctr4 overexpression (Ctr4 oe) strains. Strains whose extracts were used for the protein transformations in the meiosis experiments (Figure 5) are indicated with asterisks. **(C)** Wild-type (wt), *ctr4*Δ single and *ctr4*Δ* ctr5*Δ double mutants, and Ctr4 overexpressing cells were spotted in serial dilutions on EMM plates (Control, left) or in equal quantities onto EMM plates containing a gradient of 0 to 2.5 mM H_2_O_2_ (right).

**Figure 5:**
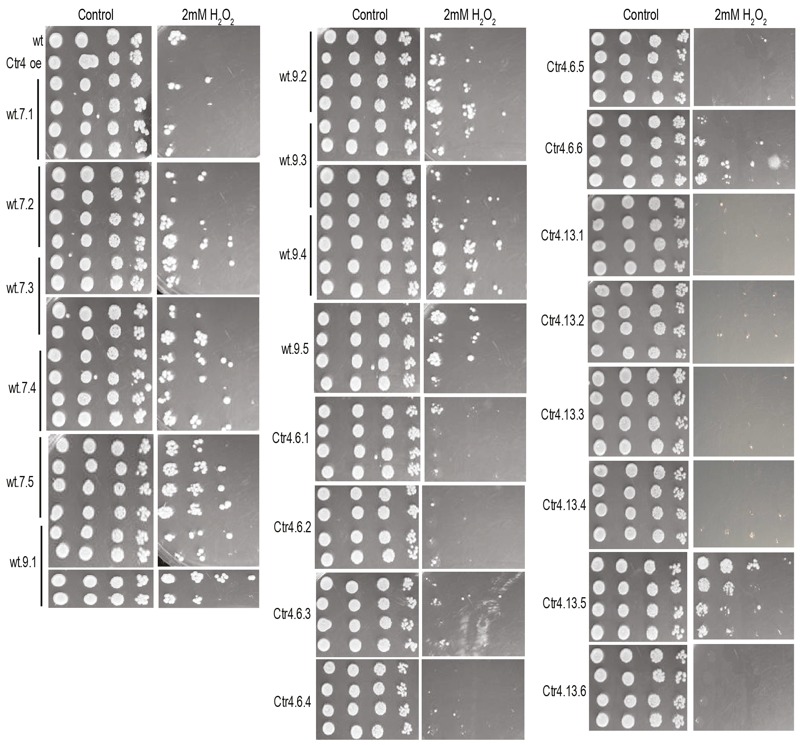
FIGURE 5: Sensitivity to H_2_O_2_ due to Ctr4 overexpression is inherited in a non-Mendelian manner. Wild-type (wt) cells transformed with extract from wild-type or Ctr4 overexpressing cells (indicated with asterisks in Figure 4) were mated with wild-type cells. The four segregants from 10 tetrads involving wild-type transformants and from 12 tetrads involving Ctr4 transformants were spotted in serial dilutions onto YES plates without (control) and with 2 mM H_2_O_2_. Five or six full tetrads from each cross involving the wt.7, wt.9, Ctr4.6, Ctr4.13 transformants were examined. All plates shown have been grown together for the same times and using the same batches of media.

To quantify the effects of oxidative stress, we first determined the ratio of the maximum growth rates for the overexpression strain in H_2_O_2 _vs control medium. This ratio decreased ~4.5 to 7-fold for cells overexpressing Ctr4 compared to the wild-type cells (**Figure 4A**), suggesting that Ctr4 overexpression leads to increased sensitivity to oxidative stress. This phenotype was retained even after the cells were grown in 3 mM GdnHCl for at least 30 generations (data not shown). The H_2_O_2 _sensitivity of cells overexpressing Ctr4 was also confirmed by determining the viability of exponentially growing cultures exposed to H_2_O_2_ for 24 h (**Figure 4B**). In this assay, wild-type cells showed viabilities ranging from 74.7-89.7% compared with 62.2-82.0% for the Ctr4 overexpressing cells. Furthermore, serial dilution spotting assays on agar plates with and without H_2_O_2_ also revealed sensitivity to oxidative stress for cells overexpressing Ctr4 (**Figure 5**).

The increased sensitivity to oxidative stress upon overexpression of Ctr4 suggests that Ctr4 is inactivated under this condition. In *S. pombe, *high-affinity copper uptake is carried out by a heteromeric complex of Ctr4 and Ctr5 62. We therefore tested whether *ctr4*Δ single and *ctr4*Δ* ctr5*Δ double mutants also showed sensitivity to oxidative stress. As *ctr4*Δ mutants grew very slowly on YES medium, we performed this assay on EMM medium, on which the Ctr4 overexpressing cells grew somewhat slower (**Figure 4C**, left). Both the *ctr4*Δ single and *ctr4*Δ* ctr5*Δ double mutants and the Ctr4 overexpressing cells showed increased sensitivity to oxidative stress compared to wild-type cells (**Figure 4C**, right). This result indicates that Ctr4 overexpression leads to loss of Ctr4 function.

A key property of any prion-mediated phenotype is that it can be transmitted to naïve cells by transfer of the altered conformational form. We therefore investigated whether the increased sensitivity to H_2_O_2 _in cells overexpressing Ctr4 was transmissible to other cells using protein transformation. Cell-free extracts were prepared from wild-type and Ctr4 overexpressing cells and high molecular weight ‘insoluble’ fractions of these extracts co-transformed into wild-type naïve cells as described in Materials and Methods. The growth of eleven independently-derived transformed colonies in the presence of H_2_O_2 _was evaluated alongside a control strain and a strain overexpressing Ctr4 (**Figure 4A**). Eight out of 15 wild-type colonies transformed with Ctr4 overexpression extracts became more sensitive to H_2_O_2_ compared to wild-type colonies transformed with wild-type extracts. Such protein transformation with *S. cerevisiae* prions rarely generates >50% prion transformants 20. Similar results were obtained using the viability assay: transformation with extracts from cells overexpressing Ctr4 typically led to increased sensitivity to oxidative stress as shown in **Figure 4B**. Thus, wild-type cells transformed with an insoluble fraction of cells overexpressing Ctr4, which contains high molecular weight polymers of Ctr4 resistant to SDS and PK (**Figure 3**), often become sensitive to H_2_O_2_. We therefore conclude that the phenotype caused by Ctr4 overexpression is transmissible between cells, consistent with Ctr4 acting as a prion.

### Ctr4-dependent sensitivity to oxidative stress is inherited in a non-Mendelian manner

Yeast prions and other cytoplasmic genetic determinants are inherited in a non-Mendelian manner during meiosis. Our finding that the sensitivity to H_2_O_2_ caused by Ctr4 overexpression can be transmitted to wild-type cells by protein transformation is consistent with this feature. To directly check for non-Mendelian inheritance, we examined two strains, designated Ctr4.6 and Ctr4.13 that showed the sensitivity to H_2_O_2_ phenotype and two control strains, wt.7 and wt.9 (**Figure 4**, asterisks). We mated each of these four strains with the wild-type strain and dissected four spores from the resulting tetrads. Each resulting spore clone was tested for sensitivity to H_2_O_2 _by serial dilution on agar plates. Five or six tetrads from each cross were tested (**Figure 5**). Analysis of the products of meiosis for both crosses involving the Ctr4.6 and Ctr4.13 strains revealed an increased H_2_O_2_ sensitivity in the majority of tetrads (10 out of the 12 tetrads examined), resulting in all four spores showing the H_2_O_2_ sensitive phenotype. The control strains largely resulted in spore colonies showing H_2_O_2_ sensitivities similar to wild-type (**Figure 5**). These results show that the increased sensitivity to oxidative stress due to Ctr4 overexpression segregates in a non-Mendelian manner, to all four spores resulting from a meiotic division. Moreover, the results confirm that meiotic products that do not contain the integrated Ctr4-YFP construct, can nevertheless maintain the Ctr4-specific phenotype. This finding is expected for a cytoplasmically-transmitted trait and is characteristic of prions in budding yeast. We designate this transmissible element at [*CTR*^+^] in keeping with the nomenclature used for *S. cerevisiae *prions.

## DISCUSSION

Mammalian prions can cause neurodegenerative diseases, whereas fungal prions can be detrimental, beneficial or have no apparent impact on the host cell 2,63,64. It is likely that prions are more widespread than currently appreciated, and that they can act as protein-based epigenetic elements allowing cells to acquire new traits in specific conditions such as stress. So far, prions have only been identified in two fungal species (*S. cerevisiae*, *P. anserina*) and in mammals, although there is recent evidence to suggest that they may also exist in plants 3. Although a prion-like state has been reported in fission yeast that allows cells to survive in the absence of the essential chaperone calnexin, the responsible protein(s) that determine this phenotype remain to be identified 65. So far no other prion-like epigenetic determinants have been reported in fission yeast.

*S. pombe,* which is only remotely related to *S. cerevisiae,* encodes the full repertoire of the molecular chaperones that are required for prion propagation in *S. cerevisiae,* and as we show here, can form and propagate the *S. cerevisiae *[*PSI*^+^] prion. Thus, *S. pombe* contains the molecular machinery required for the formation and propagation of this heterologous prion. On the other hand, a previous study has shown that expression of the *S. pombe *Sup35 N-terminal region fused to the *S. cerevisiae *C-terminal domain of Sup35 does not lead to [*PSI*^+^] formation in *S. cerevisiae *66, suggesting that the prion-forming ability of Sup35 is not conserved in *S. pombe* or that the prion-forming domain is not at the N terminus of the protein.

In a proteome-wide screen, we have identified the *S. pombe* Ctr4 protein that, when overexpressed, can form a heritable, conformationally distinct protein with all of the required characteristics of a prion that leads to a trait we have designated as [*CTR*^+^]. Ctr4 normally functions as a subunit for a copper transporter complex 62,67,68,69, and deletion of *ctr4 *has been associated with sensitivity to the iron chelator ferrozine and to the DNA damaging agents 4-nitroquinoline N-oxide and hydroxyurea 55,70.

Ctr4 is predicted to contain a PrD according to the PLAAC algorithm 49, and this PrD coincides with the highest predicted unfolded (disordered) region according to the DISOPRED3 algorithm 51. To test the ability of Ctr4 to switch to and propagate as a prion we applied criteria used for *S. cerevisiae* prions. First, overexpression of Ctr4 as an YFP fusion resulted in Ctr4 clusters and ribbon-like patterns at the cell periphery, in contrast to Ctr4-GFP expressed at lower levels which localizes more evenly to the cell periphery 55. Second, overexpression of Ctr4 resulted in the formation of pelletable aggregates that were resistant to exposure to both detergent (SDS) and protease (proteinase K). Third, overexpression of Ctr4 resulted in a H_2_O_2 _sensitivity phenotype that could be transmitted to naïve cells by protein transformation. Fourth, the H_2_O_2 _sensitivity [*CTR*^+^] phenotype was inherited in a non-Mendelian manner during meiosis in crosses with naïve [*ctr*^-^] cells, a behaviour also seen with prions in *S. cerevisiae*.

Conflicting evidence exists in the literature about the effectiveness of *S. pombe* Hsp104 in facilitating budding yeast prion propagation 41,42. We find that GdnHCl abolished ScSup35 aggregate formation and prion ‘infectivity’ in *S. pombe,* suggesting that the *S. pombe* Hsp104 can propagate *S. cerevisiae* [*PSI*^+^]. On the other hand, *S. pombe *Hsp104 was not required for maintenance and propagation of the [*CTR*^+^] prion. This result raises the question that if Hsp104 is not required for the propagation of [*CTR*^+^], what chaperones are? There is at least one prion in *S. cerevisiae* which does not require Hsp104, i.e. [*GAR^+^*] which leads to cellular resistance to glucose-associated repression of alternative carbon sources 37. In the case of [*GAR*^+^], one of the Hsp70 family of chaperones (Ssa1) is absolutely required for its propagation 37,71, while members of the Hsp40 and Hsp70 families are known to contribute to the propagation of various other yeast prions 72. It remains to be established which *S. pombe* chaperone(s) - if any - are required to propagate [*CTR*^+^] in *S. pombe.* Notably, a recent study reports that ~1% of the budding yeast proteins can exert prion-like patterns for inheriting biological traits; these proteins are non-amyloid but feature large intrinsically disordered domains, and the transmission of many of these proteins does not depend on Hsp104 73,74.

Why might [*CTR*^+^] formation cause sensitivity to oxidative stress? Ctr4 is a high affinity copper transporter, and copper is an essential co-factor for enzymes involved in critical cellular processes including protection from oxidative stress 55. Most prions lead to loss of function for the corresponding proteins 2, and it is therefore plausible that the [*CTR*^+^] prion compromises the supply of sufficient copper which is required for enzymes involved in the oxidative stress response. Consistent with this view, we find that deletion of *ctr4 *also leads to increased sensitivity to oxidative stress.

Among the 295 *S. pombe *proteins with predicted PrDs, other promising candidates include Sol1, Cyc8 and Sup35, each of which has a *S. cerevisiae* orthologue that forms prions 2. Several *S. pombe* nucleoporins similar to the *S. cerevisiae* Nup100 prion 75 also contain a PrD. On the other hand, while there is no direct orthologue of Ctr4 in *S. cerevisiae*, neither of the two *S. cerevisiae* proteins that share domains with Ctr4, i.e. Ctr1 and Ctr3, show any prion-like sequences or properties. Moreover, unlike for *S. pombe *Ctr4, a DISOPRED3 analysis 51 did not reveal any extended stretches of disordered regions for *S. cerevisiae *Ctr1 or Ctr3 (not shown). It is likely that *S. pombe* contains more prion-forming proteins. By further investigating the biology of prions in fission yeast, we will be able to gain new insights into prion function, both beneficial and detrimental, and their evolution. The Ctr4-based [*CTR*^+^] prion identified here is a first step towards establishing *S. pombe *as a model system for this unique form of protein-based inheritance which may be much more widespread than suggested by the low number of species in which prions have been described and studied so far.

## MATERIALS AND METHODS

### Yeast strains, plasmids, and growth conditions

The *S. pombe* reference strain of the Bioneer deletion collection, ED668 *h^+^ ade6-M216 ura4-D18 leu1-32 *76 was used to study overexpression of *S. cerevisiae* Sup35 (using the *S. pombe nmt1* promoter on the pREP41 vector in the absence of thiamine 77) and for protein transformations. *S. cerevisiae* [*PSI*^+^] and [*psi*^-^] strain derivatives of 74D-694 (*MATa ade1-14 trp1-289 his3-200 ura3-52 leu2-3,112,* [*PIN^+^*]) were used 48. The *972 h^-^ S. pombe *strain was used for genetic crosses. The Ctr4-YFP overexpression strain was obtained from the overexpression ORFeome library 54. The *SUP35 *ORF was amplified from the [*psi*^-^] *S. cerevisiae* strain and cloned into the *Bam*HI and *Sma*I cloning sites of the pREP41-GFP vector. The *hsp104 *gene was deleted in *S. pombe *using the *natMX6* cassette 78. Tetrads were dissected with a Singer MSM 400 micromanipulator. The *ctr4*Δ single mutant (*h+ his7-366 leu1-32 ura4-D18 ade6-M210 ctr4*Δ*:ura4+*) and *ctr4*Δ* ctr5*Δ double mutant *(h+ his7-366 leu1-32 ura4-D18 ade6-M210 ctr4*Δ*:ura4+ ctr5*Δ*:KANr*), along with the wild-type control strain FY435 (*h+ his7-366 leu1-32 ura4- *Δ*18 ade6-M210*) were a kind gift from Dr Simon Labbé.

Yeast extract with supplements (YES) medium was used for most experiments, except for certain conditions, e.g. to maintain plasmids in growing cells or to support growth of *ctr4*Δ mutants, where Edinburgh minimal medium (EMM), with supplements if indicated, was used instead. For oxidative-stress sensitivity assays, we spotted 5-fold serial dilutions of 2 x 10^5^ cells onto EMM plates (control) and spotted 2 x 10^5^ cells across EMM plates containing 0 to 2.5 mM H_2_O_2_, obtained by combining slanted EMM agar and EMM agar plus 2.5 mM H_2_O_2_ (**Figure 4C**; all these EMM media contained adenine, uracil, leucine and histidine supplements), or spotted four 10-fold serial dilutions (OD_600_ 1, 0.1, 0.01, 0.001) onto YES plates with or without 2 mM H_2_O_2 _(**Figure 5**). Alternatively, growth was monitored in a Biolector microfermentor as previously described 79, using 1 mM H_2_O_2_ and exponential phase cultures set to OD_600_ ~0.15 at the start of the experiment (**Figure 4A**). Viability was determined after exponential phase cultures were diluted to OD_600_ 0.003, with or without adding H_2_O_2_ to 0.5 mM; after incubation for 24 h at 32°C, cells were plated onto YES agar (**Figure 4B**). To eliminate prions, single colonies were successively streaked onto three subsequent YES plates containing 3 mM GdnHCl. Single colonies were then picked from the last plate for experimental analyses.

### Protein analyses 

Protein extraction and subcellular fractionation were performed as described previously 58, with some modifications. Exponential phase cultures (40 ml) were centrifuged at 4000 rpm for 3 min to collect cell pellets which were washed once in 1 ml lysis buffer containing 10 mM potassium buffer pH 7.5, 250 mM NaCl, 2 mM PMSF, 1 tablet/10 ml mini protease inhibitor cocktail (Roche). Cell pellets were re-suspended in 400 µl of lysis buffer, and 50 µl glass beads (0.5 mm, Sigma) were added to break cells in a bead beater for five 40 sec cycles with samples being left on ice for 2 min between cycles. Cell debris was removed by centrifugation at 5000 rpm for 5 min at 4°C, and the supernatant (‘total protein extract’) was centrifuged at 20,000 x g for 45 min to separate soluble from insoluble fractions. The pellets were then re-suspended in 60 µl of lysis buffer (‘insoluble fraction’) for dot blot analysis (5 µl spotted on to nitrocellulose membrane) and for protein transformations (20 µl used). For proteinase K (PK) treatment, 2 or 5 µg of PK was added to 30 µl of total protein extract and incubated at 37°C for 30 min. To terminate the PK reaction, 5 mM of PMSF was added, and samples were run on SDS-PAGE for western blotting using standard protocols. Analysis of Ctr4 aggregates by SDD-AGE was performed as described before 57. For all western blots, an anti-GFP antibody (Santa Cruz Biotechnology) was used at 1:2000 and an anti-Cdc2 antibody (Sigma) at 1:5000, incubating overnight at 4°C, followed by incubation with anti-rabbit or anti-mouse antibodies (Abcam), respectively, at 1:5000 for 1 h at room temperature.

### Protein transformation

For transformation of proteins into fission or budding yeast, a standard protocol was used 80, and 20 µl of insoluble protein fraction was prepared as described above, containing 0.025 units/µl benzonase to digest any nucleic acids present in the sample. Then, 20 ml of exponential phase cell cultures were centrifuged, the cell pellets were washed and resuspended in 1 ml of 0.1 M lithium acetate (LiAc). For each transformation, we used 100 µl of cells, adding 260 µl of 40% PEG/0.1 M LiAc mix, 20 µl of insoluble cell extract prepared as above, and the pRS416 81 and pREP42 77 plasmids for *S. cerevisiae* and *S. pombe, *respectively. This transformation mix was incubated at 30°C for 1 h, after which 43 µl of pre-warmed DMSO was added, followed by heat shock at 42°C for 5 min. Pellets were collected and washed once in 1 ml of sterile water, re-suspended in 500 µl of sterile water, and 250 µl of cells were then plated on EMM agar selective medium.

## SUPPLEMENTAL MATERIAL

Click here for supplemental data file.

All supplemental data for this article are also available online at http://microbialcell.com/researcharticles/the-copper-transport-associated-protein-ctr4-can-form-prion-like-epigenetic-determinants-in-schizosaccharomyces-pombe/.

## References

[1] Prusiner SB (1982). Novel proteinaceous infectious particles cause
scrapie.. Science.

[2] Liebman SW, Chernoff YO (2012). Prions in yeast.. Genetics.

[3] Chakrabortee S, Kayatekin C, Newby GA, Mendillo ML, Lancaster A, Lindquist S (2016). Luminidependens (LD) is an Arabidopsis protein with prion
behavior.. Proc Natl Acad Sci USA.

[4] Imran M, Mahmood S (2011). An overview of human prion diseases.. Virology J.

[5] Aguilar-Calvo P, Garcia C, Espinosa JC, Andreoletti O, Torres JM (2015). Prion and prion-like diseases in animals.. Virus Res.

[6] Schmitz M, Dittmar K, Llorens F, Gelpi E, Ferrer I, Schulz-Schaeffer WJ, Zerr I (2016). Hereditary Human Prion Diseases: an Update.. Mol Neurobiol.

[7] Morales R, Callegari K, Soto C (2015). Prion-like features of misfolded Abeta and tau
aggregates.. Virus Res.

[8] Collinge J (2016). Mammalian prions and their wider relevance in neurodegenerative
diseases.. Nature.

[9] Smethurst P, Sidle KC, Hardy J (2015). Review: Prion-like mechanisms of transactive response DNA binding
protein of 43 kDa (TDP-43) in amyotrophic lateral sclerosis
(ALS).. Neuropathol Appl Neurobiol.

[10] Pearce MM, Spartz EJ, Hong W, Luo L, Kopito RR (2015). Prion-like transmission of neuronal huntingtin aggregates to
phagocytic glia in the Drosophila brain.. Nat Comm.

[11] Aguzzi A, Rajendran L (2009). The transcellular spread of cytosolic amyloids, prions, and
prionoids.. Neuron.

[12] Polymenidou M, Cleveland DW (2012). Prion-like spread of protein aggregates in
neurodegeneration.. J Exp Med.

[13] Chernova TA, Wilkinson KD, Chernoff YO (2014). Physiological and environmental control of yeast
prions.. FEMS Microbiol Rev.

[14] Halfmann R, Alberti S, Lindquist S (2010). Prions, protein homeostasis, and phenotypic
diversity.. Trends Cell Biol.

[15] Halfmann R, Lindquist S (2010). Epigenetics in the extreme: prions and the inheritance of
environmentally acquired traits.. Science.

[16] True HL, Berlin I, Lindquist SL (2004). Epigenetic regulation of translation reveals hidden genetic
variation to produce complex traits.. Nature.

[17] True HL, Lindquist SL (2000). A yeast prion provides a mechanism for genetic variation and
phenotypic diversity.. Nature.

[18] Alberti S, Halfmann R, King O, Kapila A, Lindquist S (2009). A systematic survey identifies prions and illuminates sequence
features of prionogenic proteins.. Cell.

[19] Sideri TC, Koloteva-Levine N, Tuite MF, Grant CM (2011). Methionine oxidation of Sup35 protein induces formation of the
[PSI+] prion in a yeast peroxiredoxin mutant.. J Biol Chem.

[20] Sideri TC, Stojanovski K, Tuite MF, Grant CM (2010). Ribosome-associated peroxiredoxins suppress oxidative
stress-induced de novo formation of the [PSI+] prion in
yeast.. Proc Natl Acad Sci USA.

[21] Doronina VA, Staniforth GL, Speldewinde SH, Tuite MF, Grant CM (2015). Oxidative stress conditions increase the frequency of de novo
formation of the yeast [PSI+] prion.. Mol Microbiol.

[22] Tyedmers J, Madariaga ML, Lindquist S (2008). Prion switching in response to environmental
stress.. PLoS Biol.

[23] Dalstra HJ, van der Zee R, Swart K, Hoekstra RF, Saupe SJ, Debets AJ (2005). Non-mendelian inheritance of the HET-s prion or HET-s prion
domains determines the het-S spore killing system in Podospora
anserina.. Fungal Genet Biol.

[24] Douglas PM, Treusch S, Ren HY, Halfmann R, Duennwald ML, Lindquist S, Cyr DM (2008). Chaperone-dependent amyloid assembly protects cells from prion
toxicity.. Proc Natl Acad Sci USA.

[25] Liu Y, Wei H, Qu J, Wang J, Hung T (2011). Prefibrillar aggregates of yeast prion Sup35NM and its variant
are toxic to mammalian cells.. Neurol Sci.

[26] Summers DW, Douglas PM, Ren HY, Cyr DM (2009). The type I Hsp40 Ydj1 utilizes a farnesyl moiety and zinc
finger-like region to suppress prion toxicity.. J Biol Chem.

[27] Eaglestone SS, Cox BS, Tuite MF (1999). Translation termination efficiency can be regulated in
Saccharomyces cerevisiae by environmental stress through a prion-mediated
mechanism.. EMBO J.

[28] Holmes DL, Lancaster AK, Lindquist S, Halfmann R (2013). Heritable remodeling of yeast multicellularity by an
environmentally responsive prion.. Cell.

[29] Halfmann R, Jarosz DF, Jones SK, Chang A, Lancaster AK, Lindquist S (2012). Prions are a common mechanism for phenotypic inheritance in wild
yeasts.. Nature.

[30] Aamodt K, Abel N, Abeysekara U, Abrahantes Quintana A, Abramyan A, Adamova D, Aggarwal MM, Aglieri Rinella G, Agocs AG, Aguilar Salazar S, Ahammed Z, Ahmad A, Ahmad N, Ahn SU, Akimoto R, Akindinov A, Aleksandrov D, Alessandro B, Alfaro Molina R, Alici A, Almaraz Avina E, Alme J, Alt T, Altini V, Altinpinar S, Andrei C, Andronic A, Anelli G, Angelov V, Anson C (2010). Midrapidity antiproton-to-proton ratio in pp collisons at
sqrt[s]=0.9 and 7 TeV measured by the ALICE experiment.. Phys Rev Lett.

[31] Cai X, Chen J, Xu H, Liu S, Jiang QX, Halfmann R, Chen ZJ (2014). Prion-like polymerization underlies signal transduction in
antiviral immune defense and inflammasome activation.. Cell.

[32] Ritz AM, Trautwein M, Grassinger F, Spang A (2014). The prion-like domain in the exomer-dependent cargo Pin2 serves
as a trans-Golgi retention motif.. Cell Rep.

[33] Guerin R, Turcotte C, Leroux A, Rokeach LA (2009). The epigenetic calnexin-independent state is induced in response
to environmental changes.. FEMS Yeast Res.

[34] Byrne LJ, Cole DJ, Cox BS, Ridout MS, Morgan BJ, Tuite MF (2009). The number and transmission of [PSI] prion seeds (Propagons) in
the yeast Saccharomyces cerevisiae.. PloS ONE.

[35] Tuite MF, Serio TR (2010). The prion hypothesis: from biological anomaly to basic regulatory
mechanism.. Nat Rev Mol Cel Biol.

[36] Suzuki G, Shimazu N, Tanaka M (2012). A yeast prion, Mod5, promotes acquired drug resistance and cell
survival under environmental stress.. Science.

[37] Brown JC, Lindquist S (2009). A heritable switch in carbon source utilization driven by an
unusual yeast prion.. Genes Dev.

[38] Harrison PM, Gerstein M (2003). A method to assess compositional bias in biological sequences and
its application to prion-like glutamine/asparagine-rich domains in
eukaryotic proteomes.. Genome Biol.

[39] Chernoff YO, Lindquist SL, Ono B, Inge-Vechtomov SG, Liebman SW (1995). Role of the chaperone protein Hsp104 in propagation of the yeast
prion-like factor [psi+].. Science.

[40] Byrne LJ, Cox BS, Cole DJ, Ridout MS, Morgan BJ, Tuite MF (2007). Cell division is essential for elimination of the yeast [PSI+]
prion by guanidine hydrochloride.. Proc Natl Acad Sci USA.

[41] Senechal P, Arseneault G, Leroux A, Lindquist S, Rokeach LA (2009). The Schizosaccharomyces pombe Hsp104 disaggregase is unable to
propagate the [PSI] prion.. PloS ONE.

[42] Reidy M, Sharma R, Masison DC (2013). Schizosaccharomyces pombe disaggregation machinery chaperones
support Saccharomyces cerevisiae growth and prion
propagation.. Eukaryot Cell.

[43] Fiske M, White M, Valtierra S, Herrera S, Solvang K, Konnikova A, Debburman S (2011). Familial Parkinson's disease mutant E46K alpha-synuclein
localizes to membranous structures, forms aggregates, and induces toxicity
in yeast models.. ISRN Neurology.

[44] Wickner RB, Edskes HK, Maddelein ML, Taylor KL, Moriyama H (1999). Prions of yeast and fungi. Proteins as genetic material.. J Biol Chem.

[45] Tenreiro S, Munder MC, Alberti S, Outeiro TF (2013). Harnessing the power of yeast to unravel the molecular basis of
neurodegeneration.. J Neurochem.

[46] Fruhmann G, Seynnaeve D, Zheng J, Ven K, Molenberghs S, Wilms T, Liu B, Winderickx J, Franssens V (2016). Yeast buddies helping to unravel the complexity of
neurodegenerative disorders.. Mech Ageing Dev.

[47] Zhou P, Derkatch IL, Liebman SW (2001). The relationship between visible intracellular aggregates that
appear after overexpression of Sup35 and the yeast prion-like elements
[PSI(+)] and [PIN(+)].. Mol Microbiol.

[48] Ferreira PC, Ness F, Edwards SR, Cox BS, Tuite MF (2001). The elimination of the yeast [PSI+] prion by guanidine
hydrochloride is the result of Hsp104 inactivation.. Mol Microbiol.

[49] Lancaster AK, Nutter-Upham A, Lindquist S, King OD (2014). PLAAC: a web and command-line application to identify proteins
with prion-like amino acid composition.. Bioinformatics.

[50] Bitton DA, Schubert F, Dey S, Okoniewski M, Smith GC, Khadayate S, Pancaldi V, Wood V, Bähler J (2015). AnGeLi: A Tool for the Analysis of Gene Lists from Fission
Yeast.. Front Genet.

[51] Buchan DW, Minneci F, Nugent TC, Bryson K, Jones DT (2013). Scalable web services for the PSIPRED Protein Analysis
Workbench.. Nucleic Acids Res.

[52] Chen CY, Rojanatavorn K, Clark AC, Shih JC (2005). Characterization and enzymatic degradation of Sup35NM, a yeast
prion-like protein.. Protein Science.

[53] Silva CJ, Vazquez-Fernandez E, Onisko B, Requena JR (2015). Proteinase K and the structure of PrP(Sc): The good, the bad and
the ugly.. Virus Res.

[54] Matsuyama A, Arai R, Yashiroda Y, Shirai A, Kamata A, Sekido S, Kobayashi Y, Hashimoto A, Hamamoto M, Hiraoka Y, Horinouchi S, Yoshida M (2006). ORFeome cloning and global analysis of protein localization in
the fission yeast Schizosaccharomyces pombe.. Nat Biotechnol.

[55] Labbé S, Pena MM, Fernandes AR, Thiele DJ (1999). A copper-sensing transcription factor regulates iron uptake genes
in Schizosaccharomyces pombe.. J Biol Chem.

[56] Cohen A, Ross L, Nachman I, Bar-Nun S (2012). Aggregation of polyQ proteins is increased upon yeast aging and
affected by Sir2 and Hsf1: novel quantitative biochemical and microscopic
assays.. PloS ONE.

[57] Alberti S, Halfmann R, Lindquist S (2010). Biochemical, cell biological, and genetic assays to analyze
amyloid and prion aggregation in yeast.. Methods Enzymol.

[58] Ness F, Ferreira P, Cox BS, Tuite MF (2002). Guanidine hydrochloride inhibits the generation of prion "seeds"
but not prion protein aggregation in yeast.. Mol Cell Biol.

[59] Zenthon JF, Ness F, Cox B, Tuite MF (2006). The [PSI+] prion of Saccharomyces cerevisiae can be propagated by
an Hsp104 orthologue from Candida albicans.. Eukaryot Cell.

[60] Staniforth GL, Tuite MF (2012). Fungal prions.. Prog Mol Biol Transl Sci.

[61] Chen D, Wilkinson CR, Watt S, Penkett CJ, Toone WM, Jones N, Bähler J (2008). Multiple pathways differentially regulate global oxidative stress
responses in fission yeast.. Mol Biol Cell.

[62] Beaudoin J, Laliberte J, Labbe S (2006). Functional dissection of Ctr4 and Ctr5 amino-terminal regions
reveals motifs with redundant roles in copper transport.. Microbiology.

[63] Tuite MF (2015). Yeast prions: Paramutation at the protein level?. Semin Cell Dev Biol.

[64] Saupe SJ (2011). The [Het-s] prion of Podospora anserina and its role in
heterokaryon incompatibility.. Semin Cell Dev Biol.

[65] Turcotte C, Roux A, Beauregard PB, Guerin R, Senechal P, Hajjar F, Rokeach LA (2007). The calnexin-independent state does not compensate for all
calnexin functions in Schizosaccharomyces pombe.. FEMS Yeast Res.

[66] Edskes HK, Khamar HJ, Winchester CL, Greenler AJ, Zhou A, McGlinchey RP, Gorkovskiy A, Wickner RB (2014). Sporadic distribution of prion-forming ability of Sup35p from
yeasts and fungi.. Genetics.

[67] Beaudoin J, Thiele DJ, Labbe S, Puig S (2011). Dissection of the relative contribution of the
Schizosaccharomyces pombe Ctr4 and Ctr5 proteins to the copper transport and
cell surface delivery functions.. Microbiology.

[68] Ioannoni R, Beaudoin J, Mercier A, Labbe S (2010). Copper-dependent trafficking of the Ctr4-Ctr5 copper transporting
complex.. PloS ONE.

[69] Plante S, Ioannoni R, Beaudoin J, Labbe S (2014). Characterization of Schizosaccharomyces pombe copper transporter
proteins in meiotic and sporulating cells.. J Biol Chem.

[70] Deshpande GP, Hayles J, Hoe KL, Kim DU, Park HO, Hartsuiker E (2009). Screening a genome-wide S. pombe deletion library identifies novel genes and pathways involved in
genome stability maintenance.. DNA Repair.

[71] Jarosz DF, Lancaster AK, Brown JC, Lindquist S (2014). An evolutionarily conserved prion-like element converts wild
fungi from metabolic specialists to generalists.. Cell.

[72] Chernoff YO, Kiktev DA (2016). Dual role of ribosome-associated chaperones in prion formation
and propagation.. Curr Genet.

[73] Chakrabortee S, Byers JS, Jones S, Garcia DM, Bhullar B, Chang A, She R, Lee L, Fremin B, Lindquist S, Jarosz DF (2016). Intrinsically disordered proteins drive emergence and inheritance
of biological traits.. Cell.

[74] Tuite MF (2016). Remembering the past: a new form of protein-based
inheritance.. Cell.

[75] Halfmann R, Wright JR, Alberti S, Lindquist S, Rexach M (2012). Prion formation by a yeast GLFG nucleoporin.. Prion.

[76] Kim DU, Hayles J, Kim D, Wood V, Park HO, Won M, Yoo HS, Duhig T, Nam M, Palmer G, Han S, Jeffery L, Baek ST, Lee H, Shim YS, Lee M, Kim L, Heo KS, Noh EJ, Lee AR, Jang YJ, Chung KS, Choi SJ, Park JY, Park Y, Kim HM, Park SK, Park HJ, Kang EJ, Kim HB (2010). Analysis of a genome-wide set of gene deletions in the fission
yeast Schizosaccharomyces pombe.. Nat Biotechnol.

[77] Basi G, Schmid E, Maundrell K (1993). TATA box mutations in the Schizosaccharomyces pombe nmt1 promoter
affect transcription efficiency but not the transcription start point or
thiamine repressibility.. Gene.

[78] Sato M, Dhut S, Toda T (2005). New drug-resistant cassettes for gene disruption and epitope
tagging in Schizosaccharomyces pombe.. Yeast.

[79] Rallis C, Lopez-Maury L, Georgescu T, Pancaldi V, Bähler J (2014). Systematic screen for mutants resistant to TORC1 inhibition in
fission yeast reveals genes involved in cellular ageing and
growth.. Biol Open.

[80] Beach D, Nurse P (1981). High-frequency transformation of the fission yeast
Schizosaccharomyces pombe.. Nature.

[81] Sikorski RS, Hieter P (1989). A system of shuttle vectors and yeast host strains designed for
efficient manipulation of DNA in Saccharomyces cerevisiae.. Genetics.

