# Supplementary material for: The copper transport-associated protein Ctr4 can form prion-like epigenetic determinants in Schizosaccharomyces pombe
Source: Microb Cell. 2017 Jan 2;4(1):16–28. doi: 10.15698/mic2017.01.552 (PMC5302157; doi:10.15698/mic2017.01.552)
Supplement: Supplementary file 1 [file mic-04-016-s01.pdf]

Supplemental Table 1: 295 genes predicted by PLAAC ton contain prion-forming domains

med15	*moc3*	*ste13*	*nst1*	*prt1*	*yjl4*	*SPBC1E8.02*
its3	*set3*	*mde7*	*sep*	*sec31*	*pet2*	*SPBC685.03*
ent1	*cdk9*	*mug152*	*mug70*	*cao2*	*rfa1*	*SPBP8B7.26*
cyc8	*nup124*	*pub3*	*ent3*	*cid13*	*gef1*	*SPBC1A4.04*
sec9	*sla2*	*hrd1*	*mub1*	*yog2*	*psa3*	*SPAC17G6.11c*
sel1	*sec16*	*dbp2*	*ctf1*	*pfs2*	*png2*	*SPCC622.14*
uvi15	*orc4*	*tho2*	*mal3*	*ino80*	*reg1*	*SPAC56F8.02*
sla1	*tfg1*	*ppn1*	*rga7*	*cip2*	*srw1*	*SPBC56F2.05c*
pan1	*usp103*	*dcp2*	*pzh1*	*hap2*	*rmn1*	*SPCC622.15c*
tif471	*nup44*	*pat1*	*pfl2*	*mpd2*	*mei2*	*SPAP8A3.13c*
hap5	*cut8*	*ded1*	*pom1*	*rsv1*	*nrd1*	*SPCC4G3.12c*
ucp3	*pab1*	*fhl1*	*sre1*	*phx1*	*fft3*	*SPAC30D11.14c*
mca1	*scr1*	*seb1*	*tea1*	*mug24*	*smc2*	*SPBC1604.12*
sgt2	*lsd2*	*sup35*	*rgf3*	*cxr1*	*hal4*	*SPBC36.02c*
yap18	*cdc15*	*ppk38*	*ptc4*	*csc22*	*hhp1*	*SPBC36.01c*
sol1	*adn1*	*ynb3*	*adg3*	*yjp1*	*alf1*	*SPCC553.10*
amo1	*ycn3*	*dsk2*	*nup146*	*prz1*	*pyp2*	*SPAC23H3.15c*
nup189	*srp54*	*atf21*	*myo1*	*ybu1*	*rga4*	*SPBC16H5.12c*
yni8	*yaq9*	*slm1*	*alp7*	*pfl5*	*aah1*	*SPCC63.13*
def1	*sap49*	*sec2*	*ase1*	*rax2*	*hfl1*	*SPAC4G9.19*
msi2	*rpb1*	*spt5*	*ppk1*	*yqc1*	*pif1*	*SPBC1E8.05*
hsr1	*yfn1*	*sak1*	*scw1*	*ccr4*	*atg1*	*SPAC27E2.11c*
yhz3	*snf30*	*san1*	*ssr4*	*puf3*	*map4*	*SPBC215.13*
xrn2	*yla3*	*cki3*	*sre2*	*sec65*	*tea2*	*SPBC543.02c*
sum2	*gti1*	*cfr1*	*wsp1*	*rnf10*	*mu164*	*SPCC1322.10*
adn2	*bpb1*	*spt20*	*aly2*	*mug110*	*mug69*	
fft2	*mpe1*	*wsc1*	*cdr2*	*yh05*	*ybx1*	
naf1	*pex13*	*gaf1*	*pvg4*	*lkh1*	*fab1*	
nup45	*yhk6*	*rsv2*	*ppk29*	*smi1*	*fep1*	
snf22	*fib1*	*pcr1*	*ulp2*	*kin1*	*pub1*	
spo5	*msa1*	*atg11*	*trk2*	*ynt3*	*prr1*	
nsp1	*esc1*	*sts5*	*nup155*	*pcm1*	*ydm5*	
mac1	*nod1*	*rsc1*	*wee1*	*clr1*	*meu23*	
rec7	*vid21*	*gas1*	*git3*	*rds1*	*zas1*	
yen1	*rna15*	*cut9*	*nnk1*	*mei4*	*rfa2*	
hua1	*csh3*	*pof1*	*loz1*	*rgf1*	*swi2*	
atf1	*ctr4*	*gar1*	*rec16*	*psi1*	*rxt3*	
taf12	*tup11*	*bgs4*	*wis4*	*pst1*	*ppk5*	
adn3	*dsc1*	*gas5*	*rad4*	*rdp1*	*gar2*	
ykw3	*ran1*	*puf4*	*yof7*	*ing1*	*yhu2*	
yeyh	*hsf1*	*ste7*	*ebs1*	*chr3*	*rst2*	
yh7g	*shf1*	*cuf1*	*rec15*	*cdr1*	*him1*	
cbf12	*ye04*	*pli1*	*plb1*	*hus2*	*yif1*	
toa1	*mug80*	*ybcb*	*puf2*	*gga21*	*SPCC1235.01*	
yem8	*yag7*	*ucp8*	*csx1*	*emp24*	*SPCC584.15c*	
